# Medicine-Based Evidence in Congenital Heart Disease: How Artificial Intelligence Can Guide Treatment Decisions for Individual Patients

**DOI:** 10.3389/fcvm.2021.798215

**Published:** 2021-12-02

**Authors:** Jef Van den Eynde, Cedric Manlhiot, Alexander Van De Bruaene, Gerhard-Paul Diller, Alejandro F. Frangi, Werner Budts, Shelby Kutty

**Affiliations:** ^1^Department of Cardiovascular Sciences, KU Leuven and Congenital and Structural Cardiology, UZ Leuven, Leuven, Belgium; ^2^Blalock-Taussig-Thomas Pediatric and Congenital Heart Center, The Johns Hopkins Hospital and School of Medicine, Johns Hopkins University, Baltimore, MD, United States; ^3^Department of Cardiology III-Adult Congenital and Valvular Heart Disease, University Hospital Muenster, Muenster, Germany; ^4^Centre for Computational Imaging and Simulation Technologies in Biomedicine (CISTIB), School of Computing and Medicine, University of Leeds, Leeds, United Kingdom; ^5^Leeds Institute for Cardiovascular and Metabolic Medicine, Schools of Medicine, University of Leeds, Leeds, United Kingdom

**Keywords:** artificial intelligence, congenital heart disease, deep learning, evidence-based medicine, personalized medicine, randomized controlled trial

## Abstract

Built on the foundation of the randomized controlled trial (RCT), Evidence Based Medicine (EBM) is at its best when optimizing outcomes for homogeneous cohorts of patients like those participating in an RCT. Its weakness is a failure to resolve a clinical quandary: patients appear for care individually, each may differ in important ways from an RCT cohort, and the physician will wonder each time if following EBM will provide best guidance for this unique patient. In an effort to overcome this weakness, and promote higher quality care through a more personalized approach, a new framework has been proposed: Medicine-Based Evidence (MBE). In this approach, big data and deep learning techniques are embraced to interrogate treatment responses among patients in real-world clinical practice. Such statistical models are then integrated with mechanistic disease models to construct a “digital twin,” which serves as the real-time digital counterpart of a patient. MBE is thereby capable of dynamically modeling the effects of various treatment decisions in the context of an individual's specific characteristics. In this article, we discuss how MBE could benefit patients with congenital heart disease, a field where RCTs are difficult to conduct and often fail to provide definitive solutions because of a small number of subjects, their clinical complexity, and heterogeneity. We will also highlight the challenges that must be addressed before MBE can be embraced in clinical practice and its full potential can be realized.

## Introduction

Medicine remains both art and a science. It is the art of selecting a plan for care of individual patients, informed by what science reveals through clinical trials and observational studies on groups of patients who share their condition. The art and the science are congruent to the extent that the individual patient resembles the average subject in the scientific studies. For decades, the incongruities have been suppressed by a decision framework for patient management dominated by Evidence-Based Medicine (EBM) (1). Randomized controlled trials (RCT) are widely regarded as the best available method for accumulating medical evidence and proving treatment efficacy, and is responsible for much progress throughout cardiovascular medicine, but may lack the nuance required to successfully address complex questions. For example, in congenital heart disease (CHD), the Single Ventricle Reconstruction (SVR) trial has been a major achievement (2). Its primary publication carried the message that the right ventricle to pulmonary artery shunt (RVPAS) during the Norwood procedure resulted in better transplantation-free survival at 12 months when compared to the modified Blalock-Taussig shunt (MBTS). However, later reports made new hemodynamic observations, described neurodevelopmental outcomes more completely, and provided a deeper understanding of risk factors (3). Arguably the most important observation of the SVR trial stemmed from secondary analyses showing that depending on specific characteristics, some patients would likely benefit from the MBTS rather than the RVPAS (4). Reflecting on the broad heterogeneity of the study population, perhaps this should not be surprising.

## The Limitations of Evidence-Based Medicine

Direct applicability of EBM to patients in the real world is limited because there is never a perfect match for the RCT cohorts upon which the EBM is based (Table 1). The RCT generates evidence about “*average treatment effects*” that would apply for an “*average patient in the trial population*,” thereby potentially obfuscating important differences (5). However, as exemplified by the secondary analyses of the SVR trial, a “one-size-fits-all” approach driven by RCT results is far from ideal and can place some patients at risk for receiving suboptimal care. While RCTs study single diseases in many patients, physicians typically encounter single patients with many medical conditions. This is problematic, as Boyd et al. (6) estimated that if guidelines were to be followed in a 70 year-old woman with 3 chronic diseases and 2 risk factors, she would be prescribed 19 different doses of 12 different medicines, with 10 possible significant drug interactions. The EBM approach does not address the physician's most crucial question: “How to treat the unique patient in front of me?”

**Table 1:** Opportunities and challenges when transitioning from evidence-based medicine to medicine-based evidence.

**Evidence-based medicine**	**Medicine-based evidence**	
**Characteristics**	**Opportunities**	**Challenges**
Addresses the **average patient** in the trial population, a group of patients who share a common disease or risk factor but can be very heterogeneous in other potentially important characteristics.	Makes use of **approximate matches**, a group of patients who share the highest possible similarity with the index patient on various characteristics.	•How to define approximate matches, given the depth and high dimensionality of health data that can be collected? •What margin of variability may be tolerated for fair comparisons to be made?
Discloses **average treatment effect**, the net effect in a heterogeneous group of patients, while individual responses to treatment might differ considerably.	**Individualized prediction** of optimal decisions that take into account prior data and interactions.	•Performance and reliability of predictions might differ according to setting, and may change over time. •Models that consider many complex interactions tend to be opaque and difficult to interpret (“black box”). •Risks associated with inaccurate predictions: healthcare providers and patients must remain the final authorities for establishing acceptable risks. •Need to account for sources of bias in real-life data.
**Superficial information** including some basic demographical characteristics of the study population.	**Large depth of information**: biological, clinical, social, behavioral, and environmental data collected over a lifetime, representing the narrative of a patient's health pathway (“digital twin”).	•Electronical health records are generally limited and can be of poor quality; obtaining the complete picture of a patient's health required for effective AI will require structural changes in the ways such information is collected. •Privacy issues associated with personally identifiable information. •Ethical considerations regarding incidental findings encountered in the course of comprehensive data collection.
**Limited comparisons to other effective medications**; sometimes placebo is the only control. Furthermore, often **only helpful in guiding the choice of initial treatment**.	**Variety of treatment options and decisions** can be compared against one another among a pool of approximate matches, including therapeutic modification.	•Rare treatments might be disproportionally penalized just because of a lack of data or because they have not yet been applied for indications in patients who might optimally benefit from them. •For some decisions, available data will be scarce (especially initially), resulting in predictive uncertainty for therapeutic efficacy and safety.

*AI, artificial intelligence*.

To be clear, EBM does propose that care should be individualized considering the best available evidence and patient-specific characteristics and values. In reality, however, integrating all of these factors is difficult, with two possible poor results: (1) patients with the same condition receiving the same treatments, regardless of individual characteristics which significantly alter risks and benefits, or (2) clinicians ignoring guidelines altogether and managing patients case-by-case based on poorly informed guesses.

Several other limitations which explain the lack of direct clinical applicability of findings from RCTs are listed in the Table 1. In addition, there are well-documented RCT design challenges specific to CHD (7). First, given that the lifetime prevalence of CHD is 13 per 1,000 children and 6 per 1,000 adults (8) and there is a wide range of CHD, the pool of participants for certain trials is limited. Even within a certain type of CHD, patients might present with different degrees of anatomical and physiological complexity. This issue is further compounded by the wide age spectrum in pediatrics, which may affect treatment options and responses. In the setting of pharmacological trials, variable pharmacokinetics and pharmacodynamics might complicate the picture (9), while differences in center and surgeon volumes and practices are a common issue in interventional trials (10). As a result, reducing heterogeneity while at the same time ensuring adequate patient recruitment is a central concern in any RCT design in congenital cardiology. At the same time, high costs and logistical challenges are often involved, such that conducting trials for every possible set of patient characteristics and management decisions seems inefficient and even unfeasible.

## The Promises of Medicine-Based Evidence

As alluded to above, the evidence generated by EBM only takes into account a relatively limited set of clinical characteristics. However, a person's health or illness can be influence by a wide variety of factors. Beyond clinical information, these include the environmentome, microbiome, physiology, cell biology, proteome and metabolome, epigenome and transcriptome, and the genome (11). While genetic contributions as the cause of CHD have been the subject of many investigations, a growing body of literature has shown that copy number variations, mutations, and gene-environment interactions continue to play an active role later in the life of these patients, modifying their risk of adverse outcomes and response to interventions (12). Similarly, other “omics” have been correlated with clinical phenotype (13–15). It is suggested that these sources of information may help to further refine diagnostic precision and to establish targeted therapies that may optimize quality of life and minimize future complications in patients with CHD.

**Figure 1:**
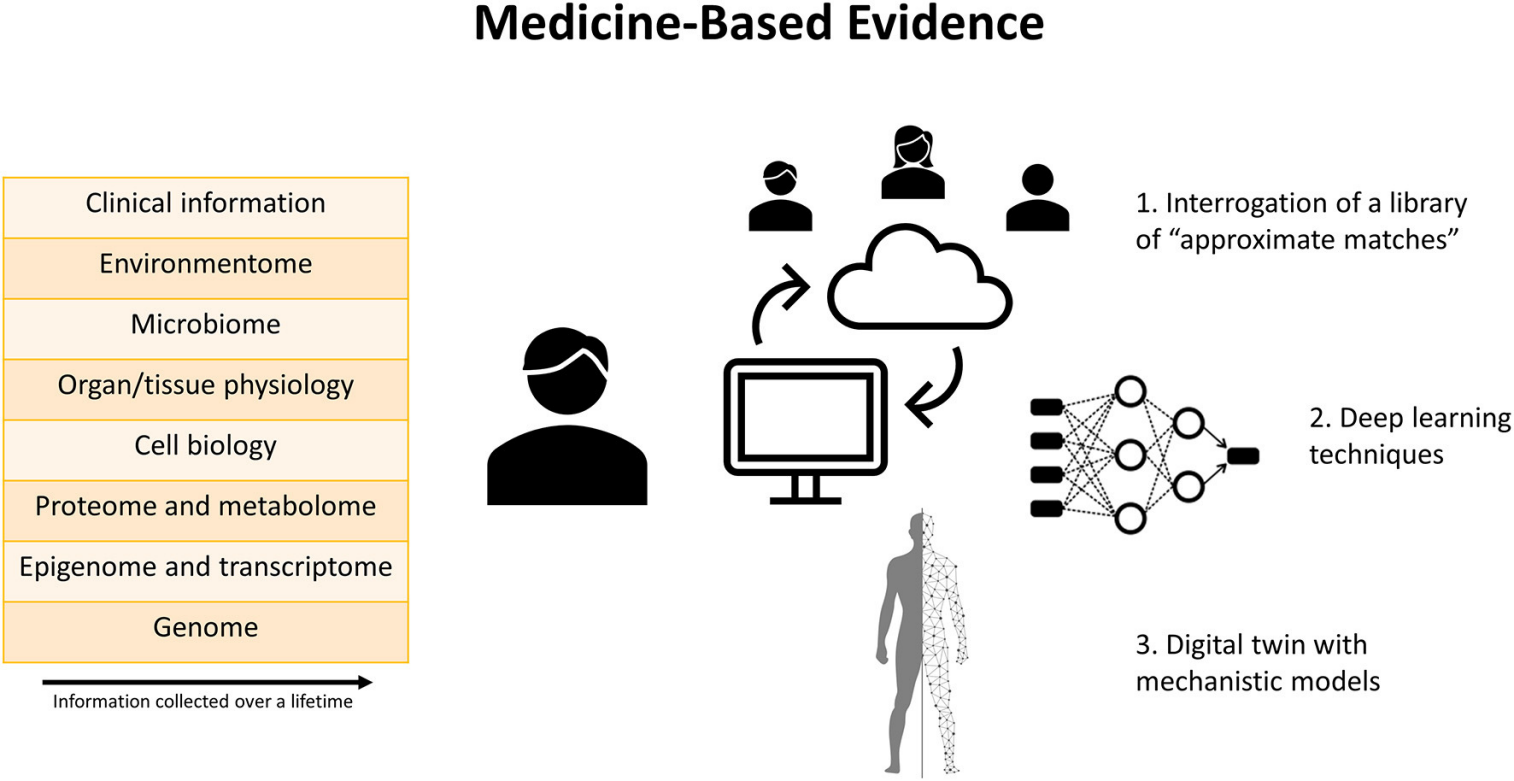
Graphical summary of the concept of Medicine-Based Evidence. Comprehensive profiling of each patient is performed based on data collected over a lifetime. Several tools are available to facilitate individualized decision-making based on these data. First, a library of “approximate matches,” consisting of a group of patients who share greatest similarity with the index case, can be interrogated to estimate the effects of various treatments within the context of the individual patient's specific characteristics. Second, deep learning techniques can detect patterns in experimental and clinical datasets from different sources. Third, a “digital twin” which incorporates mechanistic models can generate patient-level predictions according to the laws of physiology, physics, and chemistry.

In response to mounting concern about the value of EBM for decision-making, Medicine-Based Evidence (MBE) has been proposed as a means of synthetizing all available information and applying it to the individual patient (Figure 1) (16). Within this framework, big data and deep learning techniques are embraced to interrogate treatment responses among patients in real-world clinical practice. With MBE, a comprehensive profile is produced for each patient, including biological, clinical, social, behavioral, and environmental data collected over a lifetime. Whenever a physician needs to decide about a patient's treatment plan, a library of patient profiles can be interrogated. A nearest neighbor algorithm will then find *approximate matches*, a group of patients who share the greatest similarity with the index case. Some of these matches will and others will not have received the treatment under consideration, so the effect of treatment can be estimated. If a substantial majority of approximate matches has a positive response to treatment, this is evidence in favor of the treatment. Competing treatment options could be compared to see which one has the largest favorable effect. Treatment decisions may entail starting/stopping/modifying a certain medication, adding/subtracting another medication, or performing an interventional procedure. An illustrative practical example is provided in a case study of systemic lupus erythematosus by Wivel et al. (17). If large numbers of matches are unavailable, patient-level predictions using deep learning techniques could be added (18).

Rather than relying on statistical models only, a recent position paper published in the European Heart Journal suggested integrating this platform with mechanistic disease models to construct a “digital twin,” which serves as the real-time digital counterpart of a patient (19). Mechanistic models are already being widely applied for electrophysiologic and hemodynamic simulation in cardiology, including CHD (20). It is proposed that mechanistic models, harnessing our knowledge of physiology, physics, and chemistry, can complement the statistical models to enhance detection of meaningful patterns in experimental and clinical datasets. Integrating information from both types of models offers opportunities to address challenges traditionally encountered in RCTs (Table 1).

## Medicine-Based Evidence in Congenital Heart Disease

In CHD, RCTs are both difficult to conduct and commonly not definitive, so this field could potentially benefit greatly from MBE. The complexity of disease, clinical heterogeneity within lesions, and the small number of patients with specific forms of CHD severely degrade the precision and value of estimates of average treatment effects in the average patient provided by RCTs. Reflecting this, all but four recommendations in the current ESC adult CHD (ACHD) guidelines carry only a level of evidence C (21), meaning that for the large majority, no RCTs are available. Instead, the guidelines are mostly based on expert consensus and/or small studies, retrospective studies, and analyses of registries. This illustrates that currently in CHD, the idiosyncrasies of individual patients' clinical and hemodynamic characteristics are central to decision making.

MBE could have many applications in CHD. For example, prediction of late complications such as arrhythmias and congestive heart failure is difficult. Artificial intelligence (AI), especially when integrated with mechanistic models, is a very powerful means for detecting patterns and modeling the complex interactions among variables that might influence outcomes (22). Such technologies, potentially integrated with wearable monitoring devices, could provide early warning that risk of complications is increasing, alert the patient to see a physician, and suggest effective strategies to reduce risk proactively. A number of promising steps toward realizing this idea in the setting of CHD have been reported in the literature (23–26). For example, Rusin et al. (23) demonstrated that cardiorespiratory deterioration during hospitalization in patients with single ventricle could be predicted based on data from electrocardiogram and photoplethysmography. Diller et al. (24) developed an automatic deep-learning imaging algorithm that predicted death/aborted cardiac arrest or documented ventricular tachycardia in patients with tetralogy of Fallot, and in another publication (25) showed that an automatically derived disease severity score based on clinical and demographic data as well as results from electrocardiogram, cardiopulmonary exercise testing and laboratory markers could accurately predict survival in adults with CHD and effectively augment decision-making. As some have proposed, such algorithms could embrace a lifespan perspective as part of the development and implementation strategy, incorporating longitudinal data and evidence from all stages of life (27). Other potential applications include individualized prediction of the effect of drugs and/or interventions in complex hemodynamic settings (20), prediction of the feasibility of and risk associated with surgical or catheter-mediated interventions (28–32), and incorporation of “soft” outcomes such as exercise capacity and quality of life into the decision-making process (13).

## The Way Forward

This article has focused on the comparisons and contrasts between EBM and MBE, but this does not imply that the foundations of clinical practice must be based solely on one or the other. Certainly, MBE does not diminish the importance of the RCT. Instead, MBE incorporates it within a larger framework that makes use of all available evidence, whether its origins are from RCTs, observational studies, or mechanistic models. As a result, individualization of treatment would shift from today's intrinsically subjective human-driven assessment toward a more objective, data- and model-driven process that is more descriptive, integrative, and predictive (33).

While MBE has its merits and seems attractive, many hurdles to reaching its full potential lie ahead (Table 1), and it will take considerable time before MBE becomes a reality (34). First, capturing extensive data about individual patients is a major challenge. Psychological, social, and environmental factors affect health but are not always collected and organized in a useful way within a single information system. Natural language processing is being developed to extract meaningful information from electronic health records but is still in its infancy and is prone to various in the written report of a patient's health; automatically voice-captured information might present an alternative. Second, vast computational power and secure data storage and access mechanisms will be required to process the evidential database of patient profiles required for MBE. Accumulation of data will take time, but eventually, the evidence base library of MBE will grow as the medical community gathers more of the necessary data from patients across the spectrum of conditions they treat. Finally, implementation in clinical practice has been challenging, due to both data quality and privacy issues. To assure that prediction models are valid and remain so over time, AI applications should be evaluated as healthcare interventions and should continue to be monitored after approval. Collaboration between healthcare and experts in AI will be required to ensure these novel technologies can benefit our patients.

It is anticipated that the low prevalence of certain types of CHD and heterogeneity of the condition will continue to pose a challenge toward creating a substantial evidence base in congenital cardiology, at least in the initial phase. A report by the Congenital Heart Public Health Consortium (CHPHC) from 2016 mapped a complex constellation of databases with CHD data that are managed by hospitals, specialty organizations, partnerships, and public health and other governmental entities (35). Clearly, a considerable amount of infrastructure is already in place, yet issues related to accessibility, accuracy, completeness, depth, and timeliness of the collected data remain an issue. Since the publication of this report, several data collection networks for CHD have been established, including among others Advanced Cardiac Therapies Improving Outcomes Network (ACTION) (36), Fontan Outcomes Network (FON) (37), PartneRships in cOngeniTal hEart disease (PROTEA) (38), and BELgian COngenital Heart Disease Database combining Administrative and Clinical data (BELCODAC) (39). Continued efforts to establish multicenter clinical registries with in-depth and up-to-date information collection as well as meaning integration of information from various sources will be instrumental in paving the way for the full potential of MBE to be realized.

In conclusion, MBE represents a fundamental change in the way medical decisions are made. No longer seeking to identify and apply a single best approach based on the average result in an average patient, it is a major step toward true personalized medicine. This approach appears to have great potential, especially in CHD. It would, however, require professional and societal acceptance of AI as a foundation for individual medical decisions, and the support of a medical informatics and computational infrastructure which has yet to be built.

## Data Availability Statement

The original contributions presented in the study are included in the article/supplementary material, further inquiries can be directed to the corresponding authors.

## Author Contributions

JV wrote the first draft of the manuscript. CM, AV, G-PD, AF, WB, and SK critically revised the manuscript and provided important intellectual contributions. All authors contributed to manuscript revision, read, and approved the submitted version.

## Conflict of Interest

SK is consultant for GE Healthcare. G-PD is consultant for Actelion, Daiichi-Sankyo, and Bayer, previously. WB is proctor for Abbott and Occlutech. The remaining authors declare that the research was conducted in the absence of any commercial or financial relationships that could be construed as a potential conflict of interest.

## Publisher's Note

All claims expressed in this article are solely those of the authors and do not necessarily represent those of their affiliated organizations, or those of the publisher, the editors and the reviewers. Any product that may be evaluated in this article, or claim that may be made by its manufacturer, is not guaranteed or endorsed by the publisher.
